# Socio-demographic correlates of booking antenatal care in first trimester among pregnant women in Tanzania. Insights from Tanzania demographic health survey 2022

**DOI:** 10.3389/frph.2025.1669621

**Published:** 2025-10-29

**Authors:** Gladys Reuben Mahiti, Suleiman Chombo, Pankras Luoga

**Affiliations:** ^1^ Department of Development Studies, School of Public Health and Social Sciences, Muhimbili University of Health and Allied Sciences, Dar es Salaam, Tanzania; ^2^ Department of Epidemiology and Biostatistics, School of Public Health and Social Sciences, Muhimbili University of Health and Allied Sciences, Dar es Salaam, Tanzania

**Keywords:** antenatal care, pregnant women, first trimester, Tanzania, TDHS

## Abstract

**Background:**

In Tanzania, only 34% of pregnant women come for antenatal care (ANC) in first trimester hence miss an opportunity to be checked and get health advice from the health care workers. However, there is scanty of studies which used national representative data to determine the socio-demographic correlates of problem among pregnant women in Tanzania. Therefore, this study aimed at filling the gap.

**Methods:**

This study analysed secondary data involving a weighted sample size of 4,243 pregnant women aged 15–49 years from the 2022 Tanzania Demographic and Health Survey (TDHS). The analysis adapted a two-level multilevel weighted modified Poisson regression model using Stata 18 software. The dependent variable, “ANC in the first trimester of pregnancy” defined as the binary outcome variable. The independent variables included maternal factors and household factors.

**Results:**

The two-level multilevel modified Poisson regression indicated that one year increase in age of a mother at first birth results to 2% increase in prevalence of attending ANC in the first trimester (*p*-value = 0.019). Those in the richest wealth status had 31% higher prevalence of attending ANC in the first trimester compared to counterparts in poorest wealth status (*p*-value = 0.011). Those with first pregnancy had 23% higher prevalence of attending ANC in the first trimester compared to those with 24–48 months preceding birth interval (*p*-value = 0.005). Those with more than 48 months preceding birth interval had 28% higher prevalence of attending ANC in the first trimester compared to counterparts who had 24–48 months preceding birth interval (*p*-value <0.001). Those living in a household with more than 6 members had 32% less prevalence of attending ANC in the first trimester compared to those living in household with 1–4 members (*p*-value <0.001). Study participants who reported large walking distance to health facility in their residing communities indicated 24% less prevalence of attending ANC in the first trimester (*p*-value = 0.007).

**Conclusion:**

ANC attendance in the first trimester of pregnancy was significantly associated with wealth index, household size, preceding birth interval, age at first birth and distance to health facility. Programs aiming at increasing early ANC booking should prioritize activities that improve women's livelihoods, particularly those targeting lower socioeconomic and educational groups.

## Background

Globally, approximately about 300,000 women die annually from pregnancy or childbirth-caused by complications around the world and almost all of these deaths occur in low-resource settings, of which they are preventable (1). Though, statistics show maternal mortality ratio (MMR) decreased from 385 to 2016 between 1990 and 2015. But still, regional variations in terms of MMR persist; with the South Asian and the sub Saharan Africa regions bearing high burden of the global maternal and child deaths (2, 3).

The global strategy for Women's, Children's and Adolescents' Health under the Sustainable Development Goal (SDG) 3 has set the targets to reduce maternal mortality ratio (MMR) to less than 70 per 100,000 live births, and the neonatal mortality to 12 per 1,000 live births or lower by 2030 (4). The strategy includes a motive to increase Antenatal care (ANC) attendances, particularly early ANC booking in the first trimester of pregnancy.

Antenatal care (ANC) visits are crucial to reduce maternal morbidity and mortality and improve overall maternal and neonatal health outcomes. It is recommended that pregnant women start ANC in the first trimester of their pregnancy (1). The three first months give women an opportunity to be checked and get health advice from the health care workers. WHO recommends pregnant women to have eight ANC visits before delivery (5).

Globally, the coverage of early ANC visit within 14 weeks is reported to increase from 40.9% to 58.6% from the year 1990 to 2013 (6). However, the uptake rate differs between developed and developing countries, with low attendance prevalent in developing countries (6). WHO indicates late booking of ANC has negative health outcomes including death of pregnant women or her child or both (7).

The lowest levels of ANC, based on data reporting a minimum of four visits, are observed in sub-Saharan Africa and South Asia (6). The proportion of women receiving at least four ANC visits varies greatly, ranging from 13% in countries in sub-Saharan Africa to over 90% in other countries in Latin America, the Caribbean, and European regions (6). Although improvement has been recorded in the global coverage of early (starting at <12 weeks' gestation) ANC in the last two decades (8), the poorest women in LMICs often still do not have access to high-quality antenatal care (6). This situation has a number of negative health outcomes to the pregnant women and their expected new-borns (9).

Studies conducted in low and middle income countries (LMICs) (10) indicated that, overall, 49.9% of women with one or more ANC visit and 44.3% of all women had timely ANC initiation; 11.3% achieved four or more ANC visits than eight and 11.2% received no ANC. Women with timely ANC initiation had 5.2 (95% confidence interval and 4.7 times higher odds of receiving four and more ANC contacts, respectively, and were more likely to receive a higher content of ANC than women with delayed ANC initiation (10). Regionally, women in Central and Southern Asia had the best performance of timely ANC initiation; Latin America and Caribbean had the highest proportion of women achieving four to eight ANC visits. Factors associated with women not initiating ANC in the first trimester or did not achieve 8 contacts included coming for poor households, single women, with low education, living in rural areas, larger households, having short birth intervals, higher parity, and not giving birth in a health facility nor attended with a skilled attendant (10).

In Tanzania, only 34% of women book ANC services in the first trimester which in turn leads to significant decreased trend of those achieving four or more ANC visits. For example, 65%, 54.9%, 37.6% and 65% received four or more Antenatal visits in 1999, 2004/05, 2010 and 2022 (8, 11, 12). However, the coverage for the first visits as recommended by WHO is only 34% which is unacceptable (12). In the same line, there are limited studies conducted recently that used nationally representative data to analyse the factors associated with problem (13–15). Beginning ANC within the first trimester facilitates the adoption of preventive measures, the early detection of diseases, and the provision of relevant and up-to-date information. It can also encourage the integration of clinical practices, the provision of psychosocial and emotional support, the reduction of pregnancy-related complications, and the elimination of health inequalities (5, 16–18). Some of the studies in Tanzania have five years difference therefore, significance of first ANC attendance helps to reduce maternal and neonatal morbidity and mortality rate. Existing evidence indicated that the prevalence and associated factors on first ANC attendnce varied between regions and within regions with unknown causative factors within the five years of national survey. Despite using extensive data from a large population study in the country, more is needed about what constitutes ANC attendance in the first trimester of pregnancy due to the five years difference of national survey data since majority of the previous studies in the country covered small area and sample size. Therefore, this study aims to analyse the sociodemographic correlates of the ANC attendance in the first trimester among pregnant women using the 2022 Tanzania Demographic and Health Survey (TDHS) which are nationally representative data that increase strength. Studying socio demographic factors is importance as they are the key factors that influence ability of pregnant woman to book or not to book for ANC services in first trimester as recommended by the WHO and Ministry of Health of Tanzania.

## Methods

**Figure 1:**
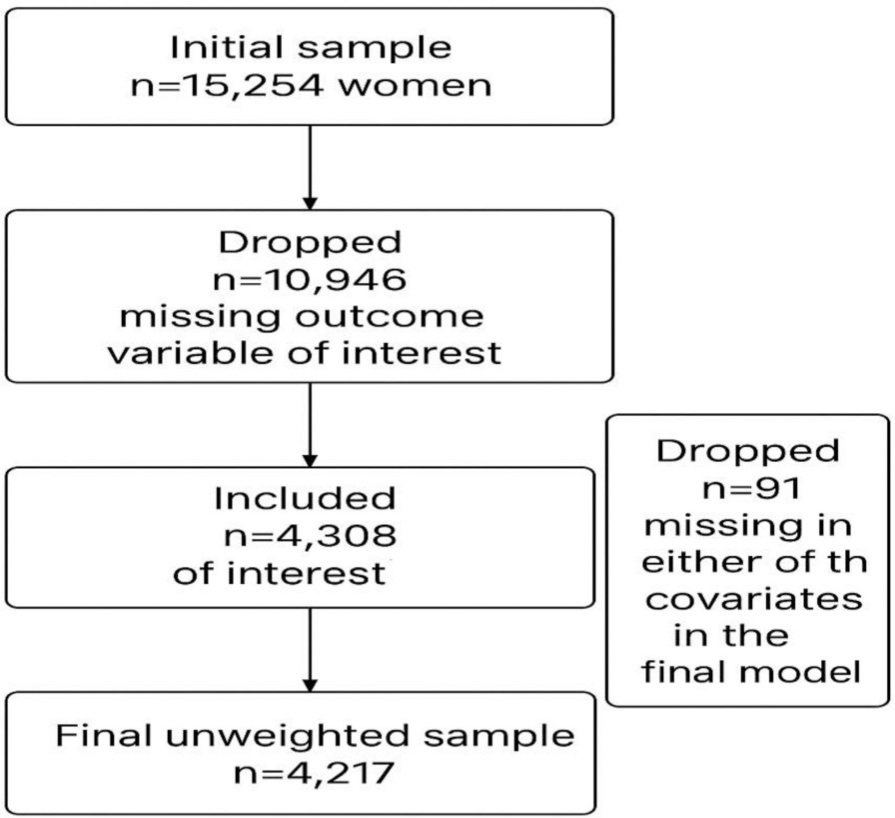
Flowchart for selected participants.

The study used cross-sectional design whereby ANC related data were collected at one point in time from each study participant. The study population comprised of pregnant women in Tanzania Demographic Health Survey, TDHS 2022. The study utilized an unweighted sample of 4,217 pregnant women drawn from all regions of Tanzania, as recorded in the 2022 TDHS (see Figure 1). To account for the complex sampling design, the sample was weighted, resulting in a final analytic sample of 4,243 participants.

The sample were obtained using two-stage cluster sampling. In the first stage, clusters were randomly selected from available sampling frame (list of available clusters) in each district from all regions of Tanzania. Then, from each selected cluster, households were randomly selected from the available list of all households within the cluster. From the selected households, women including pregnant women were sampled and included in the survey. The detailed information on the specific sampling procedures are documented elsewhere including in the final report of the 2022 TDHS (12). The survey covered various demographic and health topics including antenatal care services. This study focused on women's information regarding antenatal care. All considered variables were included in the model. Variables selection based on *p*-value criteria was not considered in model building for this study. The study used weighted multivariable modified Poisson regression model. The analysis was done using Stata 17 software. The variable, “antenatal care visits at first 3 months of pregnancy” was defined as the binary outcome variable with attributes, “yes” as 1 and “no” as 0. The independent variable in the model was termed significant if had the *p*-value less than 0.05.

Detailed description of the methodology and questionnaires used in the survey is available in the final report of the TDHS 2022 (12). The unit of analysis used was pregnant women who gave birth in two years prior the survey.

### Data management and analysis

The variables selection criteria used in this study was based on previous literature (8, 14, 19–21) important confounders and external knowledge on antenatal care visits. The variable, “antenatal care status at first 3 months of pregnancy” was defined as the binary outcome variable with attributes, “yes” as 1 and “no” as 0.

According to the research conducted by Jiwani et al. (10) and Adhikari et al. (8) on the topics of ‘Timing and number of ANC contacts in low and middle-income countries' and 'Sociodemographic correlates of antenatal care visits in Nepal,’ several key independent variables were identified. These include region (geographical zone), mother's age at first birth, residence, education level, number of household members, sex of the household head, wealth index, and preceding birth interval, all of which could be associated with ANC in the first trimester of pregnancy. Therefore, this study considered fore-mentioned variables as key variables to be considered when determining factors associated with antenatal care visits in Tanzania.

The independent variables included were based on maternal factors (age of a mother at first birth, number of children ever had (parity), education, marital status and preceding birth interval) and household factors (type of place of residence, wealth index, sex of household head as well as household size and geographical zone of household) (see Table 1). All participants with missing on the outcome variable of interest, “ANC in the first trimester” were dropped from the study. The variable, residence was removed from the final model due to high collinearity.

**Table 1:** Characteristics of study participants.

Variable	Counts (%)
Total (*n*)	4,243 (100)
Outcome variable	
Attended ANC <4 months of pregnant	
Yes	1,441 (34.0)
No	2,801 (66.0)
Proportion of ANC <4 months (95% CI)	0.34 (0.32,0.36)
Individual level variables	
Maternal factors	
Median age at first birth (IQR)	19 (17–21)
Education	
No education	881 (20.7)
Primary	2,335 (55.0)
Secondary or higher	1,027 (24.2)
Preceding birth interval	
1st child	971 (22.9)
<24 months	589 (13.9)
25–48 months	1,528 (36.0)
49+ months	1,155 (27.2)
Parity	
1	962 (22.7)
2–4	2,216 (52.2)
5+	1,065 (25.1)
Marital status	
Single	363 (8.9)
Married	2,452 (57.8)
Living with partner	1,074 (25.3)
Other	354 (8.4)
Household factors	
Residence	
Urban	1,169 (27.6)
Rural	3,074 (72.4)
Wealth index	
Poorest	966 (22.8)
Poorer	840 (19.8)
Middle	818 (19.3)
Richer	834 (19.6)
Richest	785 (18.5)
Sex of household head	
Male	3,330 (78.5)
Female	913 (21.5)
Household size	
1–4	1,303 (30.7)
5–6	1,256 (29.6)
7+	1,684 (39.7)
Zone	
Western/Lake	1,885 (44.4)
Northern/Central	876 (20.6)
Southern/Southern highlands	807 (19.1)
Eastern/Zanzibar	675 (15.9)
Cluster level variable	
Distance from health facility	
No	2,855 (67.3)
Yes	1,388 (32.7)

The study used a two-level multilevel modified Poisson regression model with a log link, estimating prevalence ratios for ANC in the first trimester. The model adjusts for multiple socio-demographic predictors, accounts for clustering at the community level (random intercepts), and allows the effect of facility distance to vary across clusters (random slopes). The multi-level model was performed using *melogit* Stata command along with *pweight* option to account for multi-stage sampling to ensure representativeness of the sample.

In the DHS dataset, the cluster identifier (v001) was used as the second-level variable. The community-level measure of distance to a health facility was derived from the individual-level variable v467d (woman's reported distance to health facility). First, a binary variable was created from v467d to indicate whether the respondent reported a “small/medium” or “large” distance. Next, cluster-level scores were computed by averaging these binary responses using the Stata command, *collapse (mean) varname, by(v001).* Finally, the averaged values were converted into a binary cluster-level indicator using the *floor(varname)* Stata function, where 0 = small/medium distance and 1 = large distance to a healthcare facility.

The dataset containing the computed second-level variable(s) was merged with the individual respondent (IR) dataset using the Stata command, *merge m:1 v001 using “second_level_dataset”*, where v001 served as the linking identifier.

The modified Poisson regression was used along with generalized multi-level model instead of Logistic regression model because the outcome of interest, “ANC visits on first trimester” was a common event [had higher prevalence more than 10% (22–24)].

Model selection was based on the Akaike Information Criterion (AIC), with the model exhibiting the lowest AIC value considered the best fitting.

**Figure 2:**
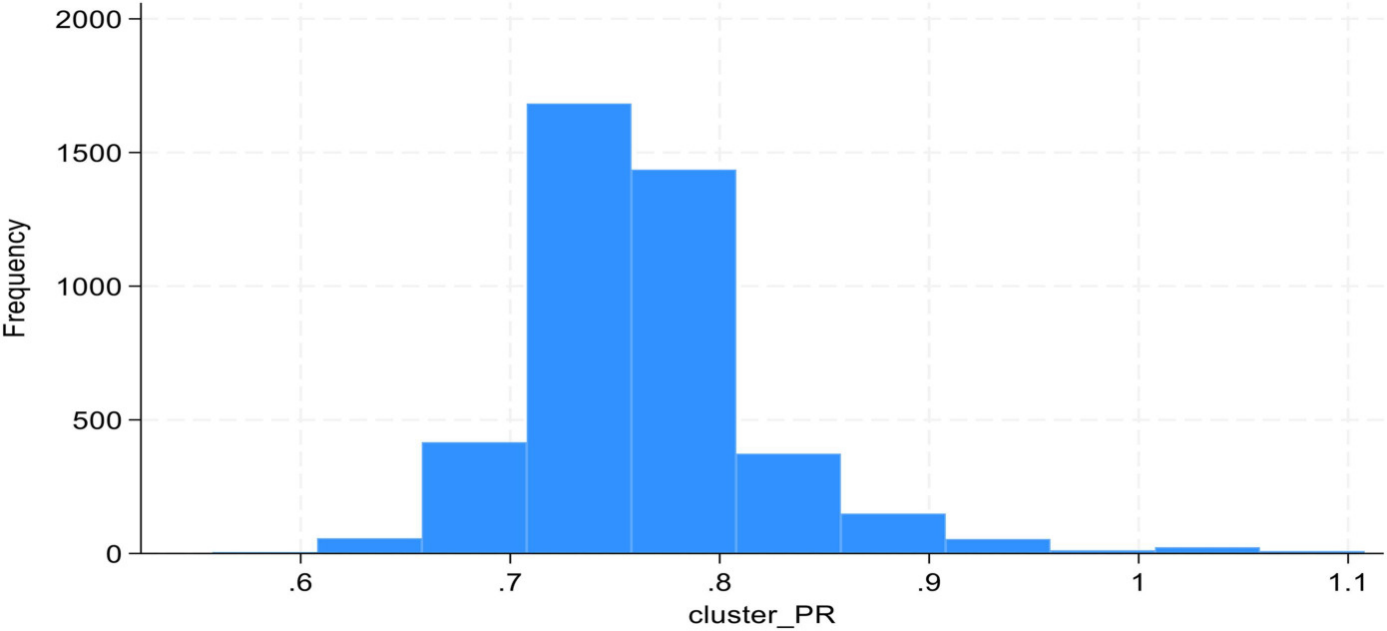
Distribution of cluster-specific PRs for distance.

The histogram was created to examine the distribution of cluster-specific prevalence ratios for second level variable (distance to health facility). The results indicated that the cluster-specific prevalence ratios for distance to health facility was normally distributed (see Figure 2).

### Ethical consideration

For this study, there was no need for ethical clearance. However, access to download data was granted by DHS custodian after reviewing our submitted concept note.

## Results

### Socio-demographic characteristics o the respondents

The results from descriptive analysis on ANC attendance on the first trimester indicated prevalence of 34% with 95% confidence intervals (32%, 36%). Participants indicated median age at first birth of 19 years with interquartile range, IQR of 4 years. Likewise, the participants reported having median number of living children of 3, with IQR of 2 children. Majority of pregnant women, 3362 (79%) had attained at least primary education level. Those reported being married were 2,452 (57.8%), while 1,074 (25.3%) reported living with partner and 363 (8.9%) were single. Of all women 971 (22.9%) reported having their first pregnancy at the time of the interview, while 589 (13.9%) had their previous pregnancy less than 24 months ago and 1,155 (27.2%) reported having their previous pregnancy 49 months ago or more.

Of all households, 913 (21.5%) had female as head of households. Likewise, 1806 (42.6%) and 818 (19.3%) households had poor wealth status and middle wealth status respectively (see Table 1).

**Figure 3:**
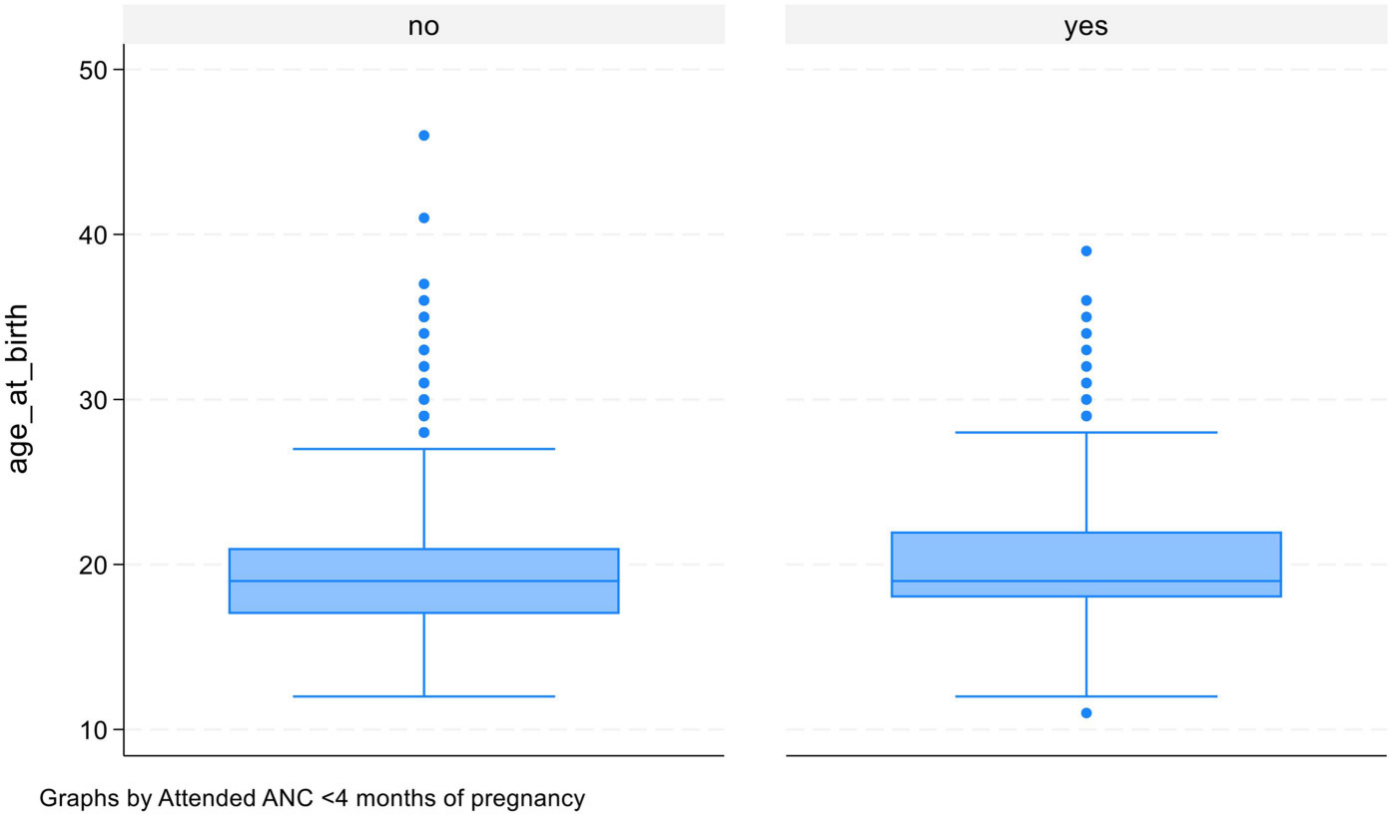
Age at first birth and antenatal care visits.

Descriptive analysis on age at first birth between those attended and not attended ANC visits in first three months. Both attended and not attended indicated median age of 19 years. But, for those attended ANC age of 18 and 22 years at first and third quartile as compared to those not attended, with age of 17 and 21 years at first and third quartile. Likewise, those attended ANC had mean age of 20 years as compared to 19 years for those not attended ANC. Therefore, the higher age at first birth could be associated with higher attendance to ANC (see Figure 3).

The results from Table 2, indicate the association based on Chi-square test of association between factor variables and ANC attendance in the first trimester of pregnancy. Residents living in rural areas indicated less attendance to ANC (31.9%) in the first trimester compared to participants living in urban areas (*p*-value = 0.0015). Pregnant women having higher education level indicated higher attendance to ANC in the first trimester as compared with participants with no education (*p*-value <0.001). ANC attendance in the first trimester was associated with individual's wealth status. Individuals with higher wealth status had higher attendance of ANC in the first trimester compared to a lower wealth status counterpart (*p*-value <0.001). Women with first pregnancy as well as women with previous pregnancy more than 48 months ago indicated higher attendance to ANC in the first trimester of pregnancy compared to women that had previous pregnancy less or equal to 48 months ago (*P*-value <0.001).

**Table 2:** Association between independent variables and antenatal care visits.

Variables	Attended ANC less than 4 months	*p*-Value
	Counts (%)	
Individual level variables		
Maternal factors		
Education		
No education (*n* = 881)	233 (26.4)	<0.001**
Primary (*n* = 2,335)	745 (31.9)	
Secondary or higher (*n* = 1,027)	464 (45.2)	
Preceding birth interval		
1st child (*n* = 971)	396 (40.8)	
<24 months (*n* = 589)	155 (26.3)	<0.001**
25–48 months (*n* = 1,528)	422 (27.6)	
49+ months (*n* = 1,155)	470 (40.7)	
Parity		
1 (*n* = 962)	388 (40.4)	<0.001**
2 to 4 (2,216)	805 (36.3)	
5+ (*n* = 1,065)	249 (23.4)	
Marital status		
Single (*n* = 363)	128 (35.2)	0.807
Married (*n* = 2,452)	844 (34.4)	
Living with partner (*n* = 1,074)	350 (32.6)	
Other (*n* = 354)	120 (33.9)	
Household factors		
Residence		
Urban (*n* = 1,169)	463 (39.6)	0.002**
Rural (*n* = 3,074)	979 (31.9)	
Wealth index		
Poorest (*n* = 966)	244 (25.3)	<0.001**
Poor (*n* = 840)	256 (30.5)	
Middle (*n* = 818)	260 (31.8)	
Richer (*n* = 834)	295 (35.4)	
Richest (*n* = 785)	387 (49.2)	
Sex of head household		
Male (*n* = 3,330)	1,132 (34.0)	0.996
Female (*n* = 913)	310 (34.0)	
Household size		
1–4 (*n* = 1,303)	561 (43.0)	<0.001**
5–6 (*n* = 1,256)	458 (36.4)	
7+ (*n* = 1,684)	424 (25.2)	
Cluster level variable		
Distance from health facility		
No	1,080 (37.8)	<0.001**
Yes	363 (26.1)	

*Indicates significance at 5% level.

After controlling for other variables, weighted multivariable modified Poisson regression results presented on Table 3 indicated education level, wealth index, household size, preceding birth interval and age at first birth to be significant independent variables associated with attending ANC services in the first trimester of pregnancy. The significance of the variable was determined at 5% level of significance.

**Table 3:** Multivariable analysis on antenatal care visits.

Variables	Univariable model		Multivariable model	
	cPR (95% Conf. interv)	*p*-Value	aPR (95% Conf. interv)	*p*-Value
Education				
No education	Ref		Ref	
Primary	1.046 (0.87–1.259)	0.633	1.025 (0.867–1.212)	0.770
Secondary or higher	1.252 (1.022–1.533)	0.030**	1.170 (0.979–1.398)	0.084
Wealth quintile				
Poorest	Ref		Ref	
Poorer	1.164 (0.977–1.387)	0.090	1.118 (0.934–1.339)	0.223
Middle	1.214 (1.018–1.447)	0.031**	1.071 (0.897–1.279)	0.446
Richer	1.308 (1.079–1.586)	0.006**	1.028 (0.838–1.261)	0.790
Richest	1.853 (1.551–2.214)	<0.001**	1.312 (1.065–1.616)	0.011**
Household size				
1 to 4	Ref		Ref	
5 to 6	0.937 (0.815–1.076)	0.355	0.887 (0.786–1.000)	0.050
7+	0.725 (0.612–0.857)	<0.001**	0.684 (0.592–0.791)	<0.001**
Preceding birth interval				
first child	1.407 (1.223–1.619)	<0.001**	1.226 (1.063–1.414)	0.005**
<24 months	0.960 (0.785–1.173)	0.688	0.979 (0.802–1.195)	0.832
24–48 months	Ref		Ref	
49+ months	1.386 (1.212–1.585)	<0.001**	1.284 (1.123–1.469)	<0.001**
Age at first birth	1.038 (1.024–1.052)	<0.001**	1.017 (1.003–1.032)	0.019**
Large distance from health facility	0.642 (0.53–0.777)	<0.001**	0.762 (0.626–0.929)	0.007**
Cluster ID (V001)				
Var (Distance from health facility)	0.247 (0.121–0.504)		0.153 (0.058–0.406)	

Ref, reference category; cPR, crude prevalence ratio; aPR, adjusted prevalence ratio. *Indicates significance at 5% level.

Pregnant women with richest wealth quintile had 32% higher prevalence of attending ANC within the first three months of pregnancy as compared to pregnant women with poorest wealth quintile [95% CI: 1.312 (1.065–1.616), *p*-value = 0.011]. Pregnant women with household size of 7 or more people indicated 32% less prevalence of attending ANC within the first trimester of pregnancy as compared to pregnant women with household size less than 5 people [95% CI: 0.684 (0.592–0.791), *p*-value <0.001]. Participants with first pregnancy indicated 23% higher of attending ANC within the first three months of pregnancy as compared to counterparts with preceding pregnancy between 24 and 48 months [95% CI: 1.226 (1.063–1.414), *p*-value = 0.005]. Likewise, participant with preceding pregnancy more than 48 months had 28% less prevalence of attending ANC on the first three months of pregnancy as compared to counterparts with preceding pregnancy between 24 and 48 months [95% CI: 1.284 (1.123–1.469), *p*-value <0.001]. One-year increase in age of women at first birth results to 2% increase in prevalence of attending ANC within first three months of pregnancy [95% C.I: 1.017 (1.003–1.032), *p*-value = 0.019].

On average, pregnant women who reported long walking distance to a health facility had a 24% less prevalence of attending ANC within the first three months of pregnancy [95% C.I: 0.762 (0.626–0.929), *p*-value = 0.007].

However, the effect of distance varied significantly across clusters/communities (random slope variance = 0.153, (95% CI: 0.058–0.406), indicating that in some areas distance is a much stronger barrier, while in others it is weaker or negligible. Accounting for this heterogeneity, the estimated cluster-specific risk ratios ranged from 0.354 (65% less prevalence) to 1.639 (64% higher prevalence), highlighting that in certain communities' distance may not be a barrier and could even be associated with higher ANC attendance.

Although the effect was not statistically significant at the 5% level, women with higher educational attainment exhibited a greater prevalence of attending ANC within the first three months of pregnancy compared to those with no formal education.

### Variation across clusters

**Figure 4:**
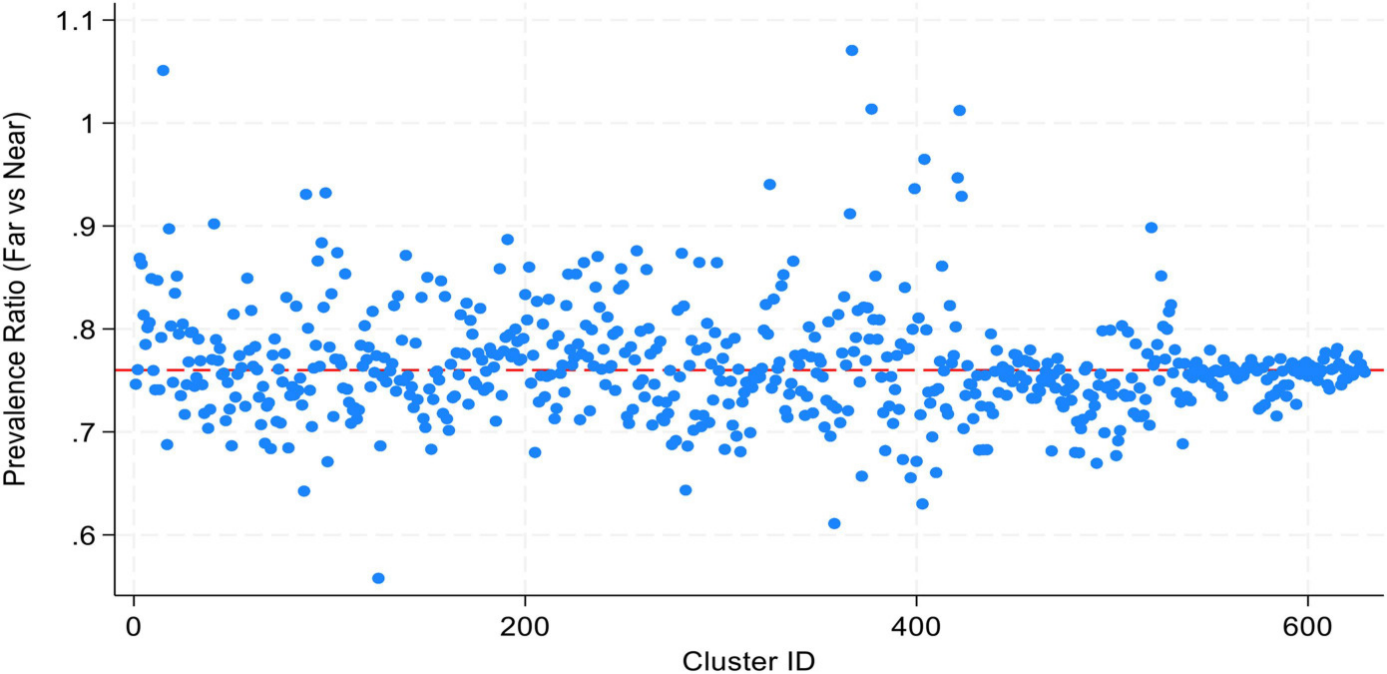
A scatter diagram for cluster-specific prevalence ratios for distance to facility.

The scatter points show that the effect of distance is not uniform. Some clusters fall well below 0.76 in these communities, being far from a facility has an even stronger negative effect (larger reduction in ANC visits). Other clusters are close to or above 1.0, in these places, distance has little or no effect, and in a few cases, being far may even be associated with higher ANC use (see Figure 4).

## Discussion

The study aimed at determining the prevalence and socio-demographic correlates of attendance to first ANC in the first trimester of pregnancy among pregnant women in Tanzania. The results show that only 34% of the women book for ANC as recommended by WHO. Our findings align with a multi-country study conducted in low middle income countries showing that less than half of women initiate ANC in the first trimester, as recommended by the World Health Organization (1, 2, 25, 26). This highlights a critical gap in maternal healthcare access and underscores the need for improved health education and outreach programs. In our setting where there are still number of challenges in healthcare services, early ANC attendance is vital for monitoring maternal and fetal health, and delays can result in adverse health outcomes. The harmful cultural norms and misconceptions surrounding pregnancy may contribute to this issue, emphasizing the need for culturally sensitive health promotion strategies.

Women with high education showed to have higher prevalence of booking ANC services in the first trimester of pregnancy compared to their counterparts with lower or no formal education. This finding implies that, ability of women in Tanzania to acquire education may improve first ANC attendance due to decision making, employment status that may increase income through income generating activities due to the number of interventions such as Tanzania Social Action Fund (TASAF) which may reduce the first and second delay in seeking health care services. Furthermore, education provides ability to gain knowledge from awareness creation through campaigns and information, education and communications materials provided to pregnant women in the community. Furthermore, existence of intervention programmes in Tanzania of increasing awareness through continuous education and the use of community health workers to make referral and providing education in the community may increase early ANC booking. Our findings are consistent with other studies (10, 20, 27–33). However, the findings are contrary to what is reported in Kigamboni Dar es Salaam, Tanzania, whereby women with tertiary education were reported to come late for ANC compared to those with secondary or below education levels (34). This may be due to their busy schedules at workplaces as high education levels may be linked to employment which is reported to result to late ANC booking (35). Strategies to increase ANC contacts must target less educated women as in most of Tanzanian communities, women with lower education lack confidence to seek early care or may not understand the significance of regular check-ups. Targeted interventions that provide health education tailored to less educated women could significantly improve early ANC booking and utilization. Addressing the educational disparities in ANC attendance requires comprehensive strategies, including enhancing access to education for girls and women, integrating maternal health education into school curricula, and using mass media to disseminate information about the importance of early ANC attendance. Also, health education programs should be culturally tailored and delivered in local languages to reach a broader audience.

Our analysis indicated that women from higher household size of seven or more were more likely to book ANC services in the first trimester of pregnancy than those coming from household with less than five. Similar findings were reported in a study conducted in Ethiopia (36). The findings imply that, a large household size may support the women in terms of household works, influencing women to seek ANC services if they happen to be influential such as mother in laws and mother of the women. Furthermore, this may be contributed by larger households that might have more resources, such as financial support, as well as support systems such as more family members available to provide support, encourage timely healthcare visits, and share responsibilities and household duties. Thus, pregnant women might have more time to prioritize their health and attend ANC appointments early.

This study also found that women from wealthier households had higher prevalence of booking ANC in the first trimester of pregnancy compared those coming from poor households. This disparity can be attributed to financial barriers faced by poorer households, which often limit their access to healthcare services. Research indicates that economic status significantly influences healthcare-seeking behaviour (11). In Tanzania, where out-of-pocket expenses for healthcare can be substantial, women from lower socio-economic backgrounds may delay seeking care due to costs. Furthermore, the findings implies that, despite the existence of the introduction of Universal Coverage Bill which was passed by Tanzania National Assembly in 2023 that has to be implemented in July 1, 2024 where it was stated a mandatory every individual to register on the health insurance care of which it can be in the formal, informal sector and the Community Health Insurance Fund still poverty may be an hindrance factor that may affect utilization of first ANC booking. Implementing financial assistance programs or subsidized services for low-income women could enhance ANC access and encourage timely attendance (8, 21, 37).

The age at first birth was a crucial factor influencing early ANC attendance. This could be due to younger mothers lack the maturity or resources to seek care effectively. In contrast, older mothers may have better access to information and healthcare services. Studies have shown that delaying childbirth is associated with improved health outcomes and increased likelihood of engaging with healthcare services including early ANC attendance (29, 37). In our society where there is high number of young women addressing the unique needs of adolescent women is critical and could positively impact maternal health. Adolescent-friendly health services that provide tailored counselling, support, and education can help young women navigate the challenges of early pregnancy and access timely ANC. Schools and community organizations should collaborate to provide comprehensive sexual and reproductive health education, empowering adolescents to make informed decisions about their health and well-being.

Interestingly, our findings suggest that women living in larger households have higher prevalence of booking ANC in the first trimester. This may be due to increased support systems in larger families which enable women to seek care more readily. Other studies have noted that social networks significantly influence health-seeking behaviour (28). In Tanzanian context, culture and familial support play a vital role in women's health decisions, and leveraging these networks could enhance the promotion of ANC attendance. The influence of social support systems on ANC attendance highlights the need to engage families and communities in maternal health initiatives. Training community health workers to conduct home visits and involve family members in ANC education can create a supportive environment for pregnant women to attend ANC in the first trimester. Additionally, involving men in maternal health programs can improve their understanding of the importance of ANC and encourage them to support their partners in seeking care.

In this study, it was noted that distance and its contribution have ambivalence contribution to the attending ANC in the first trimester of pregnancy. Some of the women who live far may have less attendance to health care services due to limitation of the long distance to the health facility. The findings are in line with studies conducted elsewhere that distance has been identified as an important barrier to the use of services, especially in rural areas (38). Studies have revealed that general health care utilization for every kind of service is affected by distance from those services. There was a decay effect of the distance on the health care service utilization, i.e., as the distance increased from the healthcare facilities; utilization of services was reduced (39). Furthermore, our findings also indicated that some women who live with long distance and short distance had poor likelihood of attending health care services. This implies the unexpected results from reported evidence that influence of the government of Tanzania to build health care facilities to the close proximity of community meaning that it will not be a barrier for utilization. Furthermore, the effect of distance may be influenced by the availability of good infrastructure and transportation within communities to the health care facilities as it was reported (38). In addition to that, the difference and dilemma may be contributed with supporting system as it was indicated that families with high household sizes have higher likelihood to utilize health care services during early ANC booking. This means distance is not a uniform barrier — some communities overcome it (e.g., via outreach services, better transport, cultural practices), while in others, distance strongly reduces ANC use.

### Strengths and limitations of the study

The study gains its strengths from using current TDHS data which provides national representative picture of the phenomenon under investigation. However, cross sectional nature of the study limited establishment of cause effect relationship between independent and dependent variables.

## Conclusion

The prevalence of ANC attendance in the first trimester among Tanzanian pregnant women is generally low at 34% compared to the WHO recommendation on first ANC. Multivariable logistic regression results indicated education level, wealth index, household size, preceding birth interval and age at first birth to be significant independent variables associated with antenatal care visits in the first trimester of pregnancy.

### Recommendation

Programs aiming at increasing ANC attendance in the first trimester pf pregnancy among pregnant mothers should prioritize activities that improve women's livelihoods through an increase of awareness and education and increasing women's education is of importance. Furthermore, use of Community Health Workers (CHWs) would be useful in strengthening the utilization of the services through referral. Not-withstanding, community involvement through alternative use of services and involvement of men could also increase utilization of ANC in the first trimester of pregnancy, particularly targeting those in lower socioeconomic and educational groups. Programme such as wealth creation and economic support among women and community may improve such utilization of the ANC services in the first trimester of pregnancy. Also, improvement of transport and infrastructure may improve utilization of early ANC booking since there was a mixed opinion regarding the distance from women's residence and health care facilities. Future research using qualitative methods is crucial to explore the underlying reasons for low ANC attendance during the first trimester of pregnancy.

## Data Availability

The datasets presented in this study can be found in online repositories. The names of the repository/repositories and accession number(s) can be found below: https://dhsprogram.com/data/dataset_admin/login_main.cfm?CFID.
